# Evobiopsychosocial medicine

**DOI:** 10.1093/emph/eoac041

**Published:** 2022-11-22

**Authors:** Adam D Hunt, Paul St-John Smith, Riadh Abed

**Affiliations:** Institute for Evolutionary Medicine, University of Zürich, Zürich, Switzerland; Evolutionary Psychiatry Special Interest Group, Royal College of Psychiatrists, London, UK; Evolutionary Psychiatry Special Interest Group, Royal College of Psychiatrists, London, UK

**Keywords:** evolutionary medicine, healthcare, biopsychosocial, education

## Abstract

The biopsychosocial model remains the de facto framework of current healthcare, but lacks causational depth, scientific rigour, or any recognition of the importance of evolutionary theory for understanding health and disease. In this article it is updated to integrate Tinbergen’s four questions with the three biopsychosocial levels. This ‘evobiopsychosocial’ schema provides a more complete framework for understanding causation of medical conditions. Its application is exemplified by tabulating depression, rheumatoid arthritis and COVID-19 within its format, which highlights the direct research and practical applications uniquely offered by evolutionary medicine. An evobiopsychosocial framework can serve as a useful tool to introduce evolutionary concepts into mainstream medicine by highlighting the broad and specific contributions of evolutionary analysis to researching, treating and preventing health conditions, providing a suitable next step for the mainstream model of medicine.

## INTRODUCTION

George Engel’s ‘biopsychosocial’ (BPS) model [1] aimed to combat excessive biomedical reductionism in medicine. Clinicians treat more than underlying pathology; they treat psychologically complex individuals within social environments. Those psychological and social factors are relevant for understanding disease and optimizing treatment. The BPS was especially welcomed by psychodynamic and socially focussed practitioners and researchers, who were losing influence to reductionistic research and treatment. It succeeded in becoming a widely acknowledged general concept of multi-dimensional healthcare, paid lip-service by various large institutions, including medical and psychiatry schools [2], but with limited effect on research direction or treatment. Approaches relying on biological reductionism have retained preference, whilst the BPS has been criticized for lacking philosophical or scientific validity [3] and being too vague, thus facilitating the choice of essentially arbitrary treatment methods and leading to difficulty in developing research agendas [2]. Recent attempts to update it [4] have not satisfied these criticisms [5].

Evolutionary medicine [6, 7] aims to connect medicine with the fundamental theory of biology. As the BPS defied biomedical short-sightedness, evolutionary medicine defies causational short-sightedness ignorant of relevant evolutionary processes. For an extended commentary on the theoretical flaws of the BPS which evolutionary approaches uniquely solve, see [8]. Of particular importance is the ability to ground judgements of function and dysfunction in naturalistic facts about adaptation [9]. This paper presents a specific expansion and enhancement of the BPS integrating evolutionary levels of analysis, illuminating why evolutionary medicine is a more complete framework for understanding health and disease, with practical implications across fields of biomedicine, clinical psychology and psychiatry, and public health.

A familiar criticism to proponents of evolutionary medicine regards its practical relevance: what does an evolutionary account have to add to treatment-as-usual, research or prevention? Specific studies or theories in evolutionary medicine often seem to have no immediate clinical implications, and are aimed at elucidating causation or particular evolutionary dynamics; this is a different goal than medicine as practiced or researched in the mainstream. Proponents of evolutionary medicine recognize the basic theory of evolutionary sciences speaks to the tasks of medicine in diagnosing, treating and preventing disease: explanatory depth matters, in myriad ways, although is not easily summarized or generalizable across conditions. One of the main aims of this paper is therefore to provide a framework to direct researchers and clinicians towards elucidating why and where explanatory depth of an evolutionary sort matters—not just in forming sound theories, but in enhancing healing. The BPS model emphasized the importance of psychosocial factors to medical practise, noting sole emphasis on the biological level leads to sub-optimal healthcare. Our aim is to enhance the BPS by emphasizing both the theoretical and practical importance of evolutionary understanding.

## TABULATING THE EVOBIOPSYCHOSOCIAL

This section introduces the Evobiopsychosocial (EBPS) schema as a tabulation, inspired by [10]. This is composed of the three BPS levels:

Biological (somatic, physical and organic systems)
Psychological (individual psychological processes and behaviours)
Social (group and environmental circumstances)

These levels are then assessed with Tinbergen’s [11] four questions:

Mechanism (immediate and ongoing direct processes)
Development (relevant predisposing history)
Function (adaptive evolutionary value)
Phylogeny (evolutionary history including multiple species)

The interactions of these levels and questions produce 12 guiding questions for analysing health and disease (Table 1), which each bring research implications and practical applications (shown applied to real examples in Tables 2, 3 and 4).

**Table 1.** Twelve EBPS questions on health and disease

Engel’s Three Bio-Psycho-Social Levels	Tinbergen’s Four Questions			
	Proximate		Ultimate/Evolutionary	
	Mechanism	Development	Function	Phylogeny
Biological	1. What are the proximate biological mechanisms?	2. What developmental processes shape the mechanism?	3. What is the function of the biological mechanisms?	4. What is the phylogeny of the biological mechanisms?
Psychological	5. What are the psychological processes of immediate importance?	6. How do the psychological processes develop for the individual?	7. In what ways are the psychological processes functional?	8. What related psychological processes occur across the phylogeny?
Social	9. What are the immediate social and environmental circumstances of importance to the condition?	10. How do the social and environmental circumstances affect development across the life course?	11. What functional reactions to social and environmental circumstances are involved?	12.How have the social and environmental circumstances affected the course of phylogeny?

**Table 2.** Example of an evobiopsychosocial analysis of depression

BPS Levels	Tinbergen’s Four Causes			
	Mechanism	Development	Function	Phylogeny
**Biological** Area of Scientific Investigation	Relevant brain circuits; receptors and genes; neurotransmitter systems’ properties (e.g. serotoninergic system) [12]	Neurological development and genetic developmental plasticity (e.g. DNA methylation modifications) [13]	Function of key molecules or hormones throughout the body (e.g. serotonin in the gut, heart and reproductive system) [14]	Shared neurotransmitter pathways and molecular mechanisms with other species (e.g. serotoninergic system in mammals) [15]
Practical Application	*Identify targets for drugs; possible discovery of biomarkers*	*Identify individuals at risk for developing condition; intervene in pathways before health problems present*	*Predict side effects of drugs and possible extraneous harms of treatment*	*Identify homologous systems in animal models for drug testing* [16]
**Psychological** Area of Scientific Investigation	Psychological processes experienced as debilitating (e.g. anhedonia, rumination, pessimism) [17]	Development and history of the individual’s problem (e.g. learned helplessness, attachment problems) [18]	Adaptive cognition dysfunction or harmful functions (e.g. low mood system overactivated; functional disengaging) [17]	Psychological and behavioural correlates in other species (e.g. low mood and subdual in primates) [19]
Practical Application	*Identify harmful thoughts, moods and behaviours as therapeutic targets*	*Pre-empt onset and intervene in harmful circumstances or psychological processes*	*Subtyping; normalize and destigmatise; inform therapy (e.g. encourage improving social standing)* [20]	*Inform therapy by observing natural triggers and alleviators of related mood states in other species*
**Social** Area of Scientific Investigation	Social triggers of the phenomenon; social perception and reaction to the phenomenon (e.g. bereavement, divorce, job loss) [21]	Lifetime and recent debilitating social environments and relationships (e.g. chronic stress, low social support) [22]	Ancestral social environments shaping functional psychological traits (e.g. hierarchy recognition and reaction, conflict reduction) [23]	Social situations which trigger analogous states in other species (e.g. hierarchy status in primates) [24]
Practical Application	*Identify scenarios suitable for short term social intervention, find depressed individuals*	*Identify areas for social reform, pre-empt at risk individuals and groups*	*Inform public health measures and social reform interventions; advise individuals on appropriate life changes*	*Identify optimum and sub-optimal social conditions in related species, directing effective social prevention and intervention*

**Table 3.** Example of an evobiopsychosocial analysis of rheumatoid arthritis

BPS Levels	Tinbergen’s Four Causes			
	Mechanism	Development	Function	Phylogeny
**Biological** Area of Scientific Investigation	Chronic inflammation involving autoantibodies and other autoimmune systems [25]	Susceptibility factors (genetic and environmental) including pattern of exposure to pathogens during early childhood [25]	Normative antibody function throughout the body [26]	Antibody system function and rheumatoid arthritis in other species [27]
Practical Application	*Identify targets for drugs and biomarkers*	*Identify individuals at risk for developing condition; intervene before health problems present*	*Predict side effects of drugs and possible extraneous harms of treatment. Identify mismatch.*	*Identify homologous systems in animal models for drug testing*
**Psychological** Area of Scientific Investigation	Pain, discomfort, low motivation, fatigue, avoidance of physical activity [28]	Process from patient noticing symptoms to seeking medical help [29]	Pain as naturally encouraging disengagement; learning and habit-forming mechanisms [30]	Behavioural reactions to pain and disability in other species [30]
Practical Application	*Identify thoughts, moods and behaviours as therapeutic targets*	*Encourage seeking medical help at an early stage*	*Reduce treatment avoidance and harmful psychological effects*	*Observe minimal level of functioning required for motivation in disabled animals*
**Social** Area of Scientific Investigation	Immediate social and employment circumstances which encourage or hinder treatment or cause distress [31]	Circumstances increasing exposure to risk factors and reducing treatment-seeking behaviours [31]	The function of seeking help or hiding distress within an ancestral social context [30]	Caring behaviour and reaction to illness within other species [30]
Practical Application	*Identify individuals least likely to seek medical support to allow early intervention*	*Identify areas for social reform which encourage prevention and intervention*	*Develop efficient campaigns to encourage help-seeking*	*Optimize social and environmental conditions to encourage help-seeking*

**Table 4.** Example of an evobiopsychosocial analysis of COVID-19

BPS Levels	Tinbergen’s Four Causes			
	Mechanism	Development	Function	Phylogeny
**Biological** Area of Scientific Investigation	Viral structure (e.g. of spike proteins); immune response [32]	Differential susceptibility; illness progression (e.g. time before symptoms); risk factors (e.g. weight, age) [33]	Viral replication mechanism; viral evolution; selection pressures [34]	Species of origin (e.g. bats and pangolin), possible non-human carriers, related coronaviruses [35]
Practical Application	*Identify biomarkers for testing; identify targets for drugs*	*Identify individuals at highest risk; intervene before exposure*	*Pre-empt and prepare for future viral morphs; develop predictive vaccinations*	*Identify suitable animal models for drug testing; study other coronaviruses to understand similarities and differences*
**Psychological** Area of Scientific Investigation	Psychological effects of infection; psychology of uninfected and previously infected individuals [36]	Individual traits affecting attitudes to viruses and vaccinations; exposure to news sources over the course of the pandemic [37]	Fear and caution as protective; mistrust of certain groups and behavioural immune system [38]	Primate and mammal behavioural responses to illness, social care and ostracization [30]
Practical Application	*Identify carriers; implement behavioural interventions*	*Identify vulnerable populations; predict areas of low public health compliance*	*Develop more effective behavioural interventions*	*Discern critical circumstances for encouraging optimal individual behaviour*
**Social** Area of Scientific Investigation	Social attitudes to pandemic and willingness to engage in protective measures [37]	Social history, culture and politics affecting pandemic responses [37]	Cooperative behaviours as functional; evolution of collective action and social norms [39]	Primate cooperation and competition dynamics [40]
Practical Application	*Identify populations at highest risk of spreading virus, direct public health measures accordingly*	*Recognize useful cultural norms to encourage; predict cultures and sub-cultures at risk*	*Identify key techniques to encourage adherence to protective measures*	*Identify circumstances which encourage or discourage society-wide cooperation*

A synthesis of Tinbergen’s and Engel’s advances, which both directed the attention of researchers and medics towards fuller understanding of relevant causation, the EBPS is provided in the same vein, aimed at extending and clarifying the model of healthcare to account for evolutionary theory. Note that the mechanism and development columns are non-evolutionary (‘proximate’ causation). These questions of current medical emphasis are expanded with wider considerations of function and phylogeny (‘ultimate’ causation). The traditionally medical turns into the traditionally evolutionary. This does not mean they are entirely separate; analysis of function and phylogeny helps make sense of mechanistic and developmental processes, as emphasized in the evolutionary behavioural sciences [41] and explored below.

Engel’s three levels recognized a hierarchy of observable material systems concurrently existing in interactive dynamics at various scales. However, causation in biology must also be understood temporally: from mechanism to individual development, to history of evolutionary function, in multiple species diverging over millions of years—clarifying this process into four questions was Tinbergen’s contribution. Emphasizing Tinbergen’s questions is a central strategy in evolutionary medicine [42].

The aim of laying out the twelve questions in the EBPS table, and the basic principle behind the evobiopsychosocial approach, is to expand modern BPS approaches with insights from evolutionary medicine. Such an EBPS schema can be used to assess any medical condition for a fuller understanding of causation, with theoretical and practical implications. Exemplified below are EPBS analyses of depression (Table 2), rheumatoid arthritis (Table 3) and COVID-19 (Table 4). Tables are followed by commentary on interesting features gained from applying the EBPS schema in the particular case. Depression requires subtyping, and COVID-19 and rheumatoid arthritis are biological conditions where psychosocial factors are hugely important—evolutionary approaches enhance understanding and response here. The use of psychiatric, physiological and infectious examples emphasizes wide applicability, also illustrating how implications differ significantly between types of condition (e.g. psychological analysis of rheumatoid arthritis concerns willingness to seek and maintain treatment; psychological analysis of depression concerns the condition itself). Insights from certain questions can generalize across multiple conditions (e.g. there are common practical applications of biological-phylogenetic analysis in identifying appropriate animal systems for therapy testing), especially of the same type of illness (e.g. infections or psychiatric conditions). Tables for the examples below are necessarily populated with only a subset of possible considerations—indeed, whole disciplines are largely situated within particular questions (e.g. biomedical research in boxes 1 and 2; evolutionary biology in 3 and 4; psychotherapy in 5 and 6; evolutionary psychology in 7 and 8; public health and epidemiology in 9 and 10; evolutionary anthropology in 11 and 12). Health professionals may be concerned with only one or several of the questions applied to a particular example or type of examples (e.g. oncologists may be interested in EBPS analyses of various cancers, child psychologists in EBPS analyses of neurodevelopmental disorders; individual interest in evolutionary insights may vary between BPS levels).

One benefit of the breadth of the EBPS tabulation (a benefit of evolutionary medicine generally) is situating a particular condition or consideration within a comprehensive and coherent theoretical framework—the framework of evolutionary theory. Within that comprehensive framework, the EBPS then makes clear the major contributions of evolutionary medicine, gained by incorporating the functional and phylogenetic analysis of the biological, psychological and social levels—current approaches concentrating solely on mechanism and development lack these insights. After exemplifying this EBPS schema and commenting on specific takeaways in the three cases, the EBPS’s advantages over the BPS and its usefulness as an applied tool for summarizing the contributions of evolutionary medicine are considered.

### Depression in the EBPS

Depression’s (Table 2) biological mechanism and development are prominent research topics within psychiatry. Despite long searches for superior anti-depressants, pharmacological intervention has hardly improved. The evolutionary expansion on the biological level of analysis might facilitate theoretically driven improvements. Side effect profiles relate to evolutionarily co-opted systems of chemicals such as serotonin, which serve multiple functional roles throughout the body; recognizing this directs attention to off-target functional systems which treatment may affect. Animal models are useful insofar as the biological system is conserved phylogenetically [16]. In depression and other psychiatric conditions, this implies models of rats and mice are often inadequate, and that mammalian relatives with more human-like brain systems, behaviour and social structure, such as primates, are more appropriate.

On the psychological and social levels, functional and phylogenetic perspectives may carry various benefits. An evolutionary explanation is potentially destigmatizing [20], and encourages novel psychosocial interventions based on an evolutionary understanding of sociality and low mood. Distinct subtypes of depression could potentially be identified [43], for example, relating to adaptive low mood in grief, social defeat or romantic difficulties. Such subtyping methods can lead to distinguishing depression as normal and potentially adaptive low mood from depression as disease. Modern environmental conditions may transform low mood into clinical depression, perhaps due to evolutionary mismatch. A deeper understanding of differing depression rates related to socioeconomic and cultural factors may arise from concentrating on novel depressogenic factors such as loneliness and lack of kin and other social support. Improved subtyping via evolutionary analysis of the psychological and social factors also offers possibilities for improved biological analysis: if depression has heterogeneous causes and manifestations which a sophisticated evolutionary analysis can parse out, sub-groups of patients may be identified with different ideal treatment regimes. Perhaps depression caused by social defeat is amenable to SSRIs, but depression caused by acute physical illness is not. Previous studies clustering subtypes together may have been inappropriate and underpowered; an evolutionary analysis separating subtypes by function could allow for better identification of biological pathways and development of personalized treatments.

One point to emphasize is how EBPS tabulations include current BPS approaches: the ‘mechanism’ and ‘development’ columns are areas of current investigation and practical application by non-evolutionary research. The columns of function and phylogeny expand them, identifying where evolutionary approaches provide additional theoretical and practical insight. Note that columns are not entirely independent from one another: improved biological-phylogenetic analysis may lead to improved drug development, a traditional practical application of investigating mechanism. Tinbergen’s questions add complementary layers of causational understanding to every level of analysis. Similarly, causation clearly occurs up and down the rows of BPS levels. Biological correlates underlie psychological reactions to social situations. Social situations affect psychological and biological processes. The psychological and social alter the biological over generations via functional selection pressures (e.g. the neural serotoninergic system is a biological system evolved partially because of its specific psychosocial effects).

Critics have claimed that Engel’s BPS pandered to psychosocial practitioners for political purposes [44]; the EBPS tabulation incorporates all levels of analysis as necessary considerations with respective relevance, justifying the inclusion of psychosocial perspectives as a matter of sound science. Different disciplines have been concentrating on different questions, with different practical applications, and fitting them together within a holistic evolutionary framework provides a more complete picture than any discipline alone. The EBPS as a conceptual framework is thus a tool of reconciliation between different approaches to healthcare. Research and practise often occurs in separate ‘camps’, disparaging of each other’s methods, and there is concern that the BPS is too permissible, allowing each practitioner free reign to ignore others and engage exclusively in their preferred approach [2, 4]. The EBPS makes interrelations between disciplines explicit, and emphasizes that different approaches can be complementary rather than competing. Perhaps the challenge of encouraging disparate fields to collaborate more closely is difficult, but evolutionary researchers should recognize their ability to be synthesizers across fields.

### Rheumatoid arthritis in the EBPS

At first thought, as an apparently straightforward bodily illness, rheumatoid arthritis (Table 3) may seem to require little input from psychosocial, let alone evolutionary, analysis. The EBPS highlights that biological function and phylogeny can inform understanding of therapeutic side effect profiles and suitable animal models, as with depression, but also provide deeper insight into cause and prevention. A functional and phylogenetic analysis may be useful to explain the apparent uniqueness of rheumatoid arthritis to humans, and significant differences in incidence across cultures and populations—where some form of mismatch appears to be implicated. Considering the biological processes involved in this autoimmune disorder through the lens of evolutionary mismatch may then direct research towards particular aspects of lifestyle or even biomedical interventions; rectifying the mismatch to prevent the disease developing.

Psychosocial factors are also recognized for their relevance. Pathological processes associated with rheumatoid arthritis may cause psychological impairment directly, or indirectly via pain and discomfort, affecting lifestyle and behaviour. Early intervention in rheumatoid arthritis is associated with improved outcomes [45]. The last two decades have seen significant improvements in treatment for rheumatoid arthritis, largely through novel drug development, especially methotrexate. Yet, whilst the disease’s progression can be slowed and pain ameliorated, it cannot be reliably reversed, and the risk of joint deformity remains.

An observation relevant to many medical conditions with clear pathophysiology and available biological treatment can be made: the importance of psychosocial analysis and intervention is in identifying vulnerable demographics, enacting public health measures, encouraging individuals to seek appropriate medical interventions and increasing treatment adherence. Biomedical approaches discover medicines, but medical treatment is chosen by psychosocial beings. This was one of Engel’s major points, which he noted in the context of a heart attack which could be ignored by a stubborn patient, to their detriment [46]. However, prior psychosocial theories have been disconnected from any evolutionary grounding.

The EBPS highlights that sickness and caring behaviours have ancient phylogenetic histories and have been shaped by historical selection pressures. Assessing pain, help-seeking and social support through an evolutionary lens lends depth of understanding to these psychosocial factors. Ancestral humans were interdependent and shared interest in group members’ health. Rituals of healing are ubiquitous in human groups, as are norms of excusing sick people from normally expected activities. Fear of sickness and negative or disgusted attitudes to sickness are common, and part of the ‘behavioural immune system’ [38]. Reluctance of sufferers to admit disability due to rheumatoid arthritis may make sense with the framing of humans as interdependent co-operators, reluctant to seem unproductive—perhaps public health messaging should emphasize medicine’s capacity to prevent functional incapacitation with early intervention. Such ideas lead to hypotheses which need testing. The generation of such novel hypotheses is a major strength of expanding psychosocial considerations with evolutionary theory.

The importance of effective psychosocial interventions may increase in the near future, as more non-communicable diseases arise from harmful lifestyles, with few effective biomedical treatments. Grounding psychosocial theories and interventions in evolutionary sciences could improve the capacity for more effective prevention as well as treatment of such conditions.

### COVID-19 in the EBPS

Biomedical research of COVID-19 (Table 4) concentrated largely on viral structure with the aim of developing treatments and tests—this was successful. However, evolutionary dynamics were inevitable, yet less well understood or prepared for. COVID-19 was the first global pandemic which could utilize sufficiently cheap novel sequencing technologies to track viral evolution in real time. Close analysis and validation of evolutionary hypotheses regarding pathogen evolution and the development of evolutionary ecology models to predict and mitigate the virus are only now possible [47]. Perhaps the key evolutionary insight is that interventions such as vaccines or lockdowns can, themselves, act as selection pressures and encourage the evolution of different viral morphs, rendering the interventions less or ineffective. The fact of disease evolution is critical additional information to guide biomedical interventions, and extrapolates across viruses, cancers and bacterial infections—the future of drug development and treatment of these conditions will benefit from recognizing that the enemy is a population which should be expected to evolve to resist our interventions, and that every treatment creates selection pressure for treatment-resistance [48]. This is a radically different view of pivotal targets of modern medicine, offering a perspective that may dramatically improve medical outcomes once sufficiently developed.

Psychosocial factors are highly significant in the spread and containment of infectious disease, especially viruses. Disparities between countries in psychosocial interventions (e.g. lockdowns, public health advice) and willingness to take vaccinations result in disparities in infections and deaths. No matter the sophistication of biotechnology in developing treatments, psychosocial beings need to accept them and curtail normal socializing to slow viral spread. Mirroring the case of rheumatoid arthritis, current mainstream strategies to encourage ideal psychosocial measures are generally uninformed by relevant evolutionary research. Understanding and predicting human responses to pandemics, lockdowns and novel treatments was thus missing theoretical depth—recognizing the functional and phylogenetic factors of importance [49, 50] may improve psychosocial interventions and save lives.

If COVID-19 had occurred within an evolutionary-medicine-aware society, responses may have been very different—viral evolution would be expected, and psychosocial interventions and messaging may have been tailored differently. The EBPS highlights the relevance of evolutionary questions in better dealing with such immediate global crises, which need depth of understanding across the biological, psychological and social levels.

## Advantages of the EBPS

The EBPS offers two main advantages: emphasizing the theoretical appeal of evolutionary medicine as a holistic framework incorporating biological science and medical research; and clearly displaying the condition-specific practical applications of evolutionary medicine, directly answering the question ‘why does an evolutionary perspective matter’.

### Theoretical appeal

Engel’s BPS was practically focussed but theoretically incomplete. The EBPS deals with its major problems, clarifying the relevance and relation of each level of analysis and contributing to causational completeness. Evolutionary medicine can take inspiration from the success of the BPS and forward itself as a suitable next iteration of the overarching framework of medicine.

Evolutionary medicine is explicitly interdisciplinary; its entire aim is to connect currently disconnected disciplines of evolutionary sciences and medicine. The BPS also pushed for a form of interdisciplinarity, but differed in its suggestions and scope—Engel was primarily concerned with medical practise rather than theoretical soundness, which appealed to psychotherapists and practitioners whose methods were steadily being overlooked, but also led to problems: the BPS was often conceived as recognizing alternative frameworks for treating or preventing a health problem, and so criticized for justifying arbitrary treatment methods [2]. The EBPS is much more specific about delineating the areas of research and practical applications of each area, incorporating them as complementary rather than competing elements. This conciliation has practical benefits in the potential to direct research agendas more effectively (e.g. in recognizing that effective preventative treatment for rheumatoid arthritis exists, but psychosocial research and intervention are now crucial) and simmering disputes over interdisciplinary research relevance. Different perspectives can be coherently aligned within the EBPS, which Engel tried to achieve for schools of psychology and sociology, without real effect on medical research agendas [2, 4]. Just as Engel recognized the value of social sciences, evolutionary medicine introduces vast fields of overlooked research, of mathematical ecological models, insights from studying primates, evolutionary anthropological perspectives on natural human health circumstances, and more; within the EBPS, their relation and relevance is clearly depicted. This acts as a rebuttal of reductionism, as the BPS attempted, but upon sounder scientific and philosophical ground.

The EBPS conveys the interdisciplinarity of evolutionary medicine as a comprehensive framework connecting BPS considerations with evolutionary explanations. It is evidently more complete than any single discipline or approach alone, and should not need full exposition to show its suitability as a theoretical framework: it can be introduced by itself, made convincing on its own merit, and lay out the approach and benefit of evolutionary medicine. The BPS’s strength was more political, appealing to numerous established healthcare professionals opposed to the increasing dominance of the biomedical approach. The strength of evolutionary medicine is primarily its theoretical soundness. This contrasting context should be clearly appreciated. The absence of a large intrinsically incentivised audience for evolutionary medicine necessitates careful consideration of the best avenue it should take into the mainstream. Most obviously, this could come by proof of practical utility.

### Practical applications and utility

In improving understanding, evolutionary medicine offers broad future improvements across diagnosis (e.g. in precise subtyping of mental disorders), treatment (e.g. in ‘adaptive therapy’ of infections and cancer to avoid treatment-resistance) and prevention (e.g. in identifying areas of mismatch for mitigation). The three examples tabulated above cover only a subset of those possibilities; any condition similarly filled in will likely find areas for improvement (e.g. in recognizing inappropriate animal models or misapprehension of psychological factors).

It is worth considering the current and future utility of the EBPS framework as a tool for medical professionals. For a specialist, the question evolutionary medicine has to answer is ‘how is this relevant to me’; as a public health researcher working on reducing obesity, or an oncologist treating prostate cancer, or a child and adolescent psychiatrist with patients with eating disorders, why should an evolutionary approach matter? Here an EBPS tabulation of a specific condition can help; the clinician is provided with an evolution-informed overview and can then concentrate on the particular boxes, rows or columns of specific interest. One oncologist might be interested in psychological reluctance to seek treatment in a particular patient; another in the evolutionary dynamics of cancer developing treatment-resistance. One eating disorder specialist might be interested in female preponderance in their clinic; another in precipitating social circumstances. The EBPS tabulation of a particular condition allows any such specific investigation, providing a depth of understanding missing in contemporary medicine. Just as psychosocial considerations may come to a clinician’s mind in specific situations with specific patients, evolutionary considerations add a specific set of tools, and will not be relevant in every case and with every patient—their utility exists in being part of the toolbox health professionals have access to, but are currently missing.

Reference materials in the form of readily available EBPS tables for a wide range of conditions would be useful in this respect. Condition-specific information is relevant for specialists and other interested parties, and EBPS tables may be easier to comprehend and browse than a review paper on evolutionary insights into a specific condition. Its framework offers an overview of the BPS factors currently accepted in mainstream medicine, whilst providing the additional evolutionary perspective—it offers an augmentation of current knowledge rather than a replacement. Of course, to populate sufficiently impressive tables regarding a variety of illnesses, much more work needs to be done.

For researchers continuing to develop the field of evolutionary medicine, the benefit of the EBPS may be even greater—not only by emphasizing the unique value and broad practical implications of evolutionary medicine, and encouraging specific investigation of functional and phylogenetic analyses across the BPS aspects of particular disorders, but in placing the field in a particular position of importance—of providing an improved framework to understand health problems, and as the next step towards a more rigorous, scientific and ultimately health-improving model of medicine. Portraying the evobiopsychosocial model as the rightful successor of the biopsychosocial may be an effective strategy to forward evolutionary medicine.
